# Mycotoxins occurrence in dry herbs used for tea preparation: method validation, analysis of bulk samples and dietary risk assessment

**DOI:** 10.1007/s12550-026-00652-2

**Published:** 2026-05-23

**Authors:** Camila Suguiura Evangelista, Denise Carvalho Mello, Eloisa Dutra Caldas, Patrícia Diniz Andrade

**Affiliations:** 1 Laboratory of Toxicology, Faculty of Health Sciences, University of Brasília, Brasilia, 70910-900 DF Brazil; 2 Faculty of Agronomy and Veterinary Medicine, University of Brasília, Brasilia, 70910-900 DF Brazil

**Keywords:** Dry herbs, Tea, Mycotoxins, Isotope-labeled internal standards, Dietary risk assessment

## Abstract

In this work, a multi-mycotoxin analytical method was validated for the determination of 15 mycotoxins and related metabolites in dry herbs commonly used for tea preparation. Samples were extracted using a modified QuEChERS procedure, and quantification was performed by UHPLC-MS/MS using matrix-matched calibration and isotope-labeled internal standards. At the lowest spiking level, recoveries ranged from 82% (AFB_1_) to 111% (FB_3_). Repeatability and intermediate precision (RSD) were below 20% for all analytes across the three fortification levels. Ninety-one samples representing 33 types of dry herbs were analyzed, of which 25.3% were positive (≥ LOQ) for at least one mycotoxin. Zearalenone (ZEN) was the most frequently detected analyte (13.2%), followed by fumonisin B_2_ (FB_2_, 4.4%), aflatoxin B_1_ (AFB_1_, 3.3%), and ochratoxin A (OTA, 3.3%). A chronic dietary risk assessment for FB_2_ and total zearalenone (tZEN = ZEN + α-zearalenol) indicated no health concern for tea consumers, with estimated intakes not exceeding 5% of the established PMTDI/TDI.

**Supplementary Information:**

The online version contains supplementary material available at 10.1007/s12550-026-00652-2.

## Introduction

A diverse range of botanical materials, including leaves, herbs, roots, flowers, seeds, bark, algae, fungi and lichen, usually in dried form, is used to prepare teas (EMA, 2006, 2010). These beverages are obtained by decoction, infusion, or maceration of one or more plant parts in water and are usually marketed in bulk form or in sachets (tea bags) (EMA, 2010). Herbal teas are used as an alternative therapy in several countries, and their diverse composition, rich in antioxidants and bioactive compounds, may contribute to improving the diets of poor nutritional quality worldwide (Gil-Serna et al. 2020; Poswal et al. 2019).

In Brazil, herbal tea refers to a product made from an authorized plant species for its preparation, whole, fragmented, or ground, with or without fermentation, toasted or not (Brazil, 2022). According to Brazilian legislation, herbal teas are regulated as food or as herbal medicines, depending on their characteristics and the intended claims. Only teas regulated as herbal medicines may claim medicinal use, because they are regulated under the same framework as traditional medicines (ANVISA, 2022). However, products with unauthorized therapeutic claims for food teas and medicinal plants are commonly found in markets and fairs and are widely consumed in the country.

Several countries have established maximum limits (MLs) for mycotoxins in teas and herbs. In the European Union, the ML for ochratoxin A (OTA) in dry herbs is 10 µg/kg (EU, 2023). Brazilian legislation requires monitoring residues and contaminants in herbs that could be used as medicinal plants or herbal medicines (Brazil, 2014). MLs are established for aflatoxin B_1_ (5 µg/kg) and total aflatoxins (20 µg/kg) in some dry herbs used as medicinal plants (Brazil, 2024a, 2024b). At the international level, Codex Alimentarius has not yet established MLs for mycotoxins in these products (CODEX, 2025).

Mycotoxigenic fungi may infect dry herbs throughout the production chain and may also be transferred to beverages prepared from contaminated herbal materials during infusion or decoction (Jai et al. 2021; Pallarés et al. 2017; Yu et al. 2022). Aflatoxins, fumonisins, ochratoxin A, trichothecenes, and zearalenone are among the most relevant mycotoxins, both for their toxicity and for their occurrence in food. Aflatoxins (AFB_1_, AFB_2_, AFG_1_ and AFG_2_) are hepatotoxic and carcinogenic, exhibiting immunosuppressive effects and impairing growth (Gemede 2025; IARC 2012). Fumonisin B_1_ has been identified as an inducer of oxidative stress and possible alterations in DNA methylation, while exposure to fumonisins (FB_1_, FB_2_ and FB_3_) has been associated with esophageal cancer and an increased risk of neural tube defects (Alizadeh et al. 2012; Anumudu et al. 2025; Gelineau-van Waes et al. 2009; Govender et al. 2025; Yu et al. 2021). FB_1_ and ochratoxin A (nephrotoxic) were classified as possible human carcinogens (IARC 1993, 2002). Exposure to deoxynivalenol (DON), an important member of the trichothecene group, can cause intestinal toxicity (acute exposure), reproductive toxicity (testicular toxicity), hepatotoxicity and nephrotoxicity (chronic exposure), whereas zearalenone (ZEN) exerts strong estrogenic and anabolic effects (Ropejko and Twarużek 2021; Zhang et al. 2024). Several authors have reported the occurrence of mycotoxins in dry herbs and infusions worldwide (Assunção et al. 2021; Caldeirão et al. 2021; Cladière et al. 2018; Zhang et al. 2022; Zhou et al. 2022a; Wan et al. 2025). Caldeirão et al. (2021) found samples contaminated with AFs and OTA in Brazil; Zhou et al. (2022a) analyzed samples from China and found the highest levels of OTA and DON; and Wan et al. (2025) found only 2 samples contaminated with FB_2_ and ZEN in Taiwan. Due to the complexity of the dry herb matrix, methods used to analyze mycotoxins in these matrices often face challenges related to extraction efficiency, low recovery, matrix effects, and high limits of quantification (LOQ) (Cho et al. 2019; Cladière et al. 2018; Reinholds et al. 2019; Zhang et al. 2023; Zhou et al. 2022b). These methods generally involve extraction with organic solvents, clean-up steps (solid phase extraction, dispersive liquid-liquid microextraction, immunoaffinity columns, QuEChERS), followed by concentration or dilution, and identification/quantification, mainly by liquid chromatography (LC) and mass spectrometry (MS) (Cho et al. 2019; Reinholds et al. 2019; Zhang et al. 2023; Zhou et al. 2022b). Although some validated methods are available, most are limited to a small number of dry herb types or restricted groups of mycotoxins (Fontana et al. 2024; Jai et al. 2021; Lu et al. 2022). Only two studies have reported the occurrence of mycotoxins in dry herbs in Brazil (Caldeirão et al. 2021; Fontana et al. 2024) and both used modified QuEChERS procedures, followed by LC-MS/MS analysis.

This study aimed to optimize and validate a method for the simultaneous analysis of aflatoxins (AFB_1_, AFB_2_, AFG_1_ and AFG_2_), citreoviridin (CTV), deoxynivalenol (DON), 15-acetyldeoxynivalenol (15-AcDON), 3-acetyldeoxynivalenol (3-AcDON), deoxynivalenol-3-glucoside (D3G), fumonisins (FB_1_, FB_2_ and FB_3_), ochratoxin A, zearalenone (ZEN) and α-zearalenol (α-ZEL) in 33 different species of dry herbs commonly used for tea preparation, using isotope-labeled internal standards and UHPLC-MS/MS.

## Materials and methods

### Chemicals and reagents

HPLC-grade acetonitrile (ACN), Supelclean™ primary secondary amine (PSA), Supelclean™ (C18), sodium chloride (NaCl, ≥ 99.5%), HPLC-grade methanol (MeOH, ≥ 99.9%) and ammonium formate (97%) were purchased from Sigma-Aldrich (St. Louis, USA); magnesium sulfate anhydrous (MgSO4) and formic acid from Supelco (Bellefonte, USA); ammonium acetate, sodium acetate anhydrous (NaOAc, 99.5%) and acetic acid (HAc) from J.T Baker (Phillipsburg, USA); sulfuric acid (> 51%) from Vetec; HPLC-grade toluene (TOL) was obtained from Mallinckrodt Baker (Phillipsburg, USA); ethyl acetate (EtAc) from Merck (Darmstadt, Germany); Graphitized Carbon Black (GCB) from Dinâmica (Indaiatuba, Brazil); ultrapure water obtained through a Milli-Q purification system from Millipore (Bedford, USA); hydrophilic PTFE syringe filters (0.45 μm pore size) from Filtrilo (Colombo, Brazil).

Standards of AFB_1_ (99.0%), AFB_2_ (99.0%), AFG_1_ (99.0%), AFG_2_ (99.5%) and d1-deoxynivalenol (d1-DON, 107.2 µg/mL, 93.3%) were obtained from Sigma-Aldrich (St. Louis, USA). CTV (97.0%) was from Enzo Life Sciences International Inc. (Farmingdale, USA). 15-AcDON (98.8%), 3-AcDON (99.4%), D3G (96.0%), DON, (98.3%), FB_1_ (98%), FB_2_ (97.9%), FB_3_ (98.5%), OTA (99.5%), ZEN (99.66%), α-ZEL, (98.7%), (^13^C_17_)-AFB_1_ (99.0%), (^13^C_17_)-AFG_1_ (99.0%), (^13^C_34_)-FB_1_ (96.3%), (^13^C_20_)-OTA (98.7%), (^13^C_18_)-ZEN (98,8%) were from Biopure (Tulln, Austria). The aflatoxins stock solutions were prepared in TOL-ACN (9:1), CVT in EtAc, OTA in TOL-HAc (99:1), and fumonisins in ACN-water (50:50), while the remaining compounds were prepared in ACN. Monthly, the concentrations of aflatoxins (AFs), OTA, ZEN, DON, 3-AcDON, 15-AcDON, and CTV were measured by UV spectrophotometry, as described by Andrade et al. (2017). A maximum variation of 3% in the estimated concentration in relation to the first check was considered acceptable. Mixed working solutions of all analytes were prepared in ACN, and all solutions were stored in amber vials at -20 ◦C. Figure S1 shows the structures of all the mycotoxins investigated in this study.

### Samples

Ninety-one bulk samples, representing 33 different dry herbs commonly used for tea preparation were purchased from retail stores and compounding pharmacies in the Federal District, Brazil (examples in Figure S2). Details of the collected samples are provided in Table S1 (Supplementary Material), including dry herbs prepared from different plant parts (bark, leaf, stalk, seed, and/or flower). Upon arrival at the laboratory, samples were stored at room temperature in the original packages, under the same conditions in which they were maintained at retail stores or compounding pharmacies. Prior to analysis, depending on the stiffness of the collected material (leaves, stems, flower stems, or bark), it was ground in a blender or knife mill and stored in polyethylene bags at room temperature until analysis.

### UHPLC–MS/MS conditions

A Shimadzu system (LC-20AD pumps, a SIL-20AD autosampler, and CTO-20AC column oven - Kyoto, Japan) coupled with a 6500 + QTRAP triple quadrupole mass spectrometer from AB SCIEX (Foster, USA) was used for the analyses. Data acquisition was performed with SCIEX OS (version 1.6.2.36627) in Selected Reaction Monitoring (SRM) mode, whereas instrument control was carried out with Analyst^®^ software (version 1.6). MS/MS parameters were optimized for each analyte by direct infusion of mycotoxin solutions (50–100 ng/mL, dissolved in MeOH/H2O) into the mass spectrometer at a flow rate of 10 µL/min. The effects of formic acid (0.1%) and ammonium formate (1 or 5 mM), or acetic acid (0.1%) and ammonium acetate (5 mM), were evaluated and the best mobile-phase additive was selected. Electrospray ionization was performed in multiple reaction monitoring (MRM) mode, operating in both positive (ESI+) and negative (ESI-) polarities. Declustering potential (DP), collision energy (CE), and collision cell exit potential (CXP) were optimized for the selected transitions at the best ESI polarity for each analyte. Ion source parameters were automatically optimized using flow injection analysis of a 75 ng/mL standard solution of the less sensitive compound in the preliminary tests, at 0.4 mL/min. Different parameters of the source were tested, including temperature (450 to 700 °C), nebulizer (GS1) and heater gas (GS2) pressures (40 to 50 psi), curtain gas (CUR) pressure (20 to 50 psi) and collision-activated dissociation (CAD) gas (high, medium and low).

Chromatographic separation was carried out with an ACQUITY UPLC BEH C18 column (30Å, 1.7 μm, 2.1 mm x 50 mm) and VanGuard ACQUITY BEH, 1.7 μm guard column, both from Waters (Milford, MA, USA). The column temperature was maintained at 40 °C, and a flow rate of 0.4 mL/min was used. The mobile phase was composed of a gradient of water (A) and methanol (B), both with the additive selected during the analyte-dependent MS/MS parameters optimization process. The gradient started at 2% B, remained constant for 1 min; increased to 20% B over 1 min, maintained for 6 min; increased to 40% B and held for 1 min; increased to 60% B in 1.5 min; increased for 70% B in 4 min; increased to 95% B in 2 min and held for 2 min. The system was equilibrated for 5 min at the initial condition between consecutive runs. Figure S3 shows an ion chromatogram containing all the mycotoxins (in-matrix).

### Extraction method optimization

A composite sample was used as a blank model matrix for extraction optimization and method validation. The selection of the plant species included in this composite sample (boldo, senna, artichoke, chamomile, “espinheira santa”, gotu kola, guarana, and passion fruit) was described by Mello et al. (2024). Most of the dry herbs used in the composite sample were prepared from leaves, but bark, flowers, stems and seeds were also included. Modified QuEChERS procedures based on Mozzaquatro et al. (2022) and Zhang et al. (2016) were tested, and conditions evaluated are shown in Table S2.

In summary, 1 g of blank material was weighed into a 50 mL Falcon tube. Samples were spiked with mycotoxin standards (concentrations ranging from 12 to 318 µg/kg) and allowed to stand for 1 h for analyte-sample equilibration. Different extraction and clean-up procedures were tested (Table S2, procedures 1 to 4), including volume of milli-Q water and standing times to ensure sample hydration, extraction with 7.5 to 15 mL acidified ACN (1 to 10% formic acid), and addition of MgSO_4_ with NaOAc or NaCl, followed by vortexing and centrifugation at 3,500 rpm for 5 min. An aliquot of the extract was transferred to a 15 mL Falcon tube containing MgSO_4_ with PSA and/or C18 and carbon graphitized black (CGB), vortexed, and centrifuged. For procedures 1 to 3, 800 µL was transferred to a vial, evaporated to dryness (Centrivap Vacuum Concentrator System, LABCONCO/Germany) and redissolved in 240 µL of MeOH: H_2_O (50:50), while for procedure 4, 1.5 mL were evaporated to dryness and redissolved in 250 µL of MeOH: H_2_O (50:50). Extracts were filtered through a 0.45 μm syringe filter and injected into the UHPLC-MS/MS. Recovery tests were performed in triplicate for each extraction and clean-up procedure. Quantification was performed using matrix-matched standard curves at concentrations ranging from 3.6 to 1000 µg/kg (5.3 to 442.5 ng/mL). Data were analyzed using GraphPad Prism 10.3.1 using two-way analysis of variance (ANOVA) followed by Tukey’s multiple comparisons test; differences were considered significant when *p* < 0.05.

### Method validation

The method that yielded the best extraction results during the optimization step was validated according to the parameters established by the Brazilian National Institute of Metrology, Quality and Technology (INMETRO, 2020). The validation procedures were carried out using a composite sample of various dried herbs, as described in the section “Extraction method optimization”.

When isotope-labeled internal calibration was used, an aliquot of 135 µL of the extract was transferred to an insert and mixed with 15 µL of the isotope internal standard working solution. The final concentration of the isotopes was: (^13^C_17_)-AFB_1_ = 3.73 ng/mL, (^13^C_17_)-AFG_1_ = 3.34 ng/mL, (^13^C_34_)-FB_1_ = 50.8 ng/mL, (^13^C_20_)-OTA = 20.08 ng/mL, (^13^C_18_)-ZEN = 16.73 ng/mL, d1-DON = 214.4 ng/mL.

To assess selectivity, fortified and non-fortified composite blank samples were injected into the UHPLC-MS/MS system to evaluate the presence of matrix interferents at the same retention time (RT) and ion ratio (IR) as the mycotoxins of interest. The *matrix effect* was estimated as the ratio of the average instrument response (areas) for matrix-matched standards and neat solution standards, evaluated for each analyte at five concentration levels, with three replicates at each level, using isotope-labeled internal and external calibration. Signal suppression or enhancement above 20% was considered an important matrix effect.

*Linearity* was assessed using the same set of samples used in the matrix effect evaluation, and the presence of outliers was verified using the Grubbs test. The linear parameters of the regression were estimated by the ordinary least squares method; the F-test tested the homogeneity of variances, and the coefficient of determination (R^2^) and significance of the regression were obtained using ANOVA. For heteroscedastic data, different weighting factors were tested (1/x, 1/x^2^, 1/y and 1/y^2^), and those with the lower sum of relative errors, with significant regressions and with no lack of fit were chosen for the regression. Calibration curves ranged from LOQ to up to 88xLOQ (ZEN). For isotope internal calibration, the relative areas (ratio between the analyte peak area and the corresponding isotope internal standard peak area) were used to obtain weighted/ordinary calibration curves.

*Recovery*, expressed as %, was evaluated by fortifying composite blank samples at 3 levels (low, intermediate and high; Table S3) with six replicates at each level. The experiment was carried out on the same day, by the same analyst and outliers were removed using the Grubbs test. Samples were quantified using in-matrix calibration curves. *Repeatability* was expressed as the relative standard deviation (%RSDr) of the replicate samples used in the recovery experiments. *Intermediate precision* was evaluated by analyzing samples fortified under the same conditions as the recovery experiments but carried out on a different day (%RSDp), except for D3G, for which only 3 replicates were performed at both intermediate and high levels due to the limited amount of standard available. LOQ was defined as the lowest level for which the method was fully validated (80 ≥ recovery ≤ 120%; RSD_r_ ≤ 20%; RSD_P_ ≤ 20%). Limits of detection (LOD) were set at the lowest concentration at which a substance could be detected.

### Dietary risk assessment

Chronic intake of mycotoxins through tea consumption was estimated using a deterministic approach, following IPCS (2020) recommendation. Given that the same person may use different dry herbs in tea preparations throughout the day, the present study grouped all dry herbs analyzed and estimated the medians and 95th percentiles (P95) of contamination levels for each mycotoxin evaluated. Non-detected samples (< LOD) were assumed to be at ½ LOD, and samples between LOD and LOQ (LOD< Samples < LOQ) were replaced by the LOD.

Exposure was assessed under two scenarios: usual tea consumption (1 cup/day) and high consumption (6 cups/day), with either a median or a high contamination level (P95). According to the manufacturer, 3–18 g of dry herb are recommended for preparing a cup of tea (200 mL; Table S1), so 3 g was used as the reference dose for the preparation. A body weight of 65 kg was assumed for the adult population. The potential risks arising from mycotoxin exposure through the consumption of tea infusions were evaluated. The estimated exposure was compared with the respective Provisional Maximum Tolerable Daily Intake (PMTDI)/ Tolerable Daily Intake (TDI) value of the compound or the sum of compounds (EFSA 2016; IPCS, 2020; JECFA, 2002).

## Results

### Optimization of UHPLC-MS/MS

The presence of formic acid (0.1%) and ammonium formate (1 or 5 mM) or acetic acid (0.1%) and ammonium acetate (5 mM) was evaluated in both positive and negative modes to select the best mobile-phase additive. Direct infusion of mycotoxin solutions in positive mode showed higher intensities of the protonated adducts [M + H]^+^, using both ammonium formate (1 and 5mM; 0.1% formic acid) or ammonium acetate (5 mM, 0.1% acetic acid) for all analytes, except for D3G, for which only the ammonium adduct was found. Higher intensities were observed for most analytes using ammonium formate (5 mM; 0.1% formic acid). In the negative mode, 3-AcDON, DON, D3G, and α-ZEL showed higher intensities using ammonium formate (5 mM; 0.1% formic acid), while 15-AcDON, OTA, and ZEN showed better results with ammonium acetate (5 mM; 0.1% acetic acid). Overall, ammonium formate (5 mM, 0.1% formic acid) was selected as a mobile-phase additive because it provided the highest intensities for most analytes tested. The protonated forms [M + H]^+^ were monitored in positive mode, and [M-H]^−^ for ZEN and α-ZEL, and [M+HCOO]^−^ for D3G were monitored in negative mode. The optimized ESI-MS/MS conditions and chromatographic parameters for mycotoxins and isotope internal standards are shown in Table S4. Ion source parameters were automatically optimized using flow injection analysis and the optimal conditions of the mass spectrometer ion source were CUR: at 30 psi, ion spray voltage at − 4500 V for ESI^−^ and 5500 V for ESI^+^, CAD at high, ion source temperature at 450 °C, GS1 at 50 psi, and GS2 at 40 psi.

### Optimization of a multi-mycotoxin modified QuEChERS extraction

**Fig. 1:**
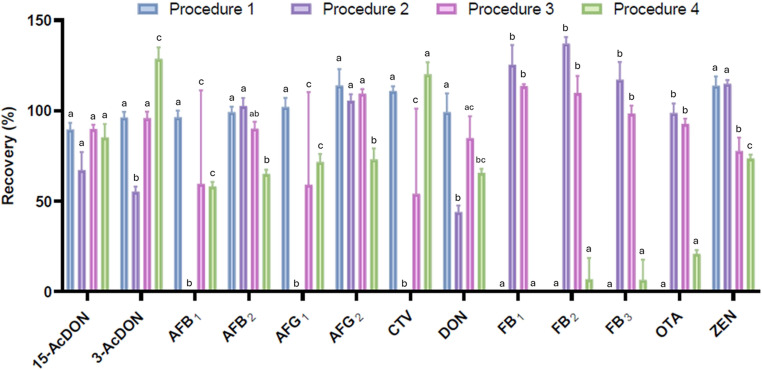
Recoveries (%) obtained using four different extraction procedures (*n* = 3), using a composite matrix and quantified using matrix-matched calibration curves (3.6 to 1000 µg/kg). Detailed information on the procedures tested is available in Table S2. Different lowercase letters indicate significant differences at *p* < 0.05 between the procedures tested for each mycotoxin

In this study, four extraction protocols were evaluated according to the methods described by Mozzaquatro et al. (2022) and Zhang et al. (2016), as well as through laboratory tests. Figure 1 shows the mean recovery rates (%) of the mycotoxins obtained using the different extraction procedures. Fumonisins (FB_1_, FB_2_ and FB_3_) and OTA were not detected when extraction was performed with 1% acidified ACN and NaOAc as a dispersion salt (Procedure 1), whereas recovery rates above 80% were obtained for the other mycotoxins. Acidifying the extraction solvent with 10% formic acid, using NaCl as the dispersion salt, and including C18 in the clean-up step (Procedure 2) yielded no recovery at the tested levels for AFB_1_, AFG_1_, and CTV, and recovery rates below 70% for 15-AcDON, 3-AcDON and DON.

In Procedure 3 (10% acidified ACN, NaCl as dispersion salt, PSA and C18 in the cleanup step), all mycotoxins showed recovery rates above 78%. In procedure 4 (C18 replaced by CGB), AFB_1_, AFB_2_, AFG_1_, AFG_2_, DON, and ZEN showed recovery rates lower than 80%, FB_1_ was not detected, FB_2_ and FB_3_ were recovered in only one replicate, and OTA also showed a recovery rate lower than 25%. The results showed that Procedure 3 performed best among the tested conditions and was selected for validation. In summary, samples were extracted with 15 mL of acidified ACN (10% formic acid), 5 g MgSO4 + NaCl (4:1; dispersion salt), 0.6 g MgSO4 + PSA + C18 (6:1:1; cleanup).

### Multi-mycotoxins method validation

**Fig. 2:**
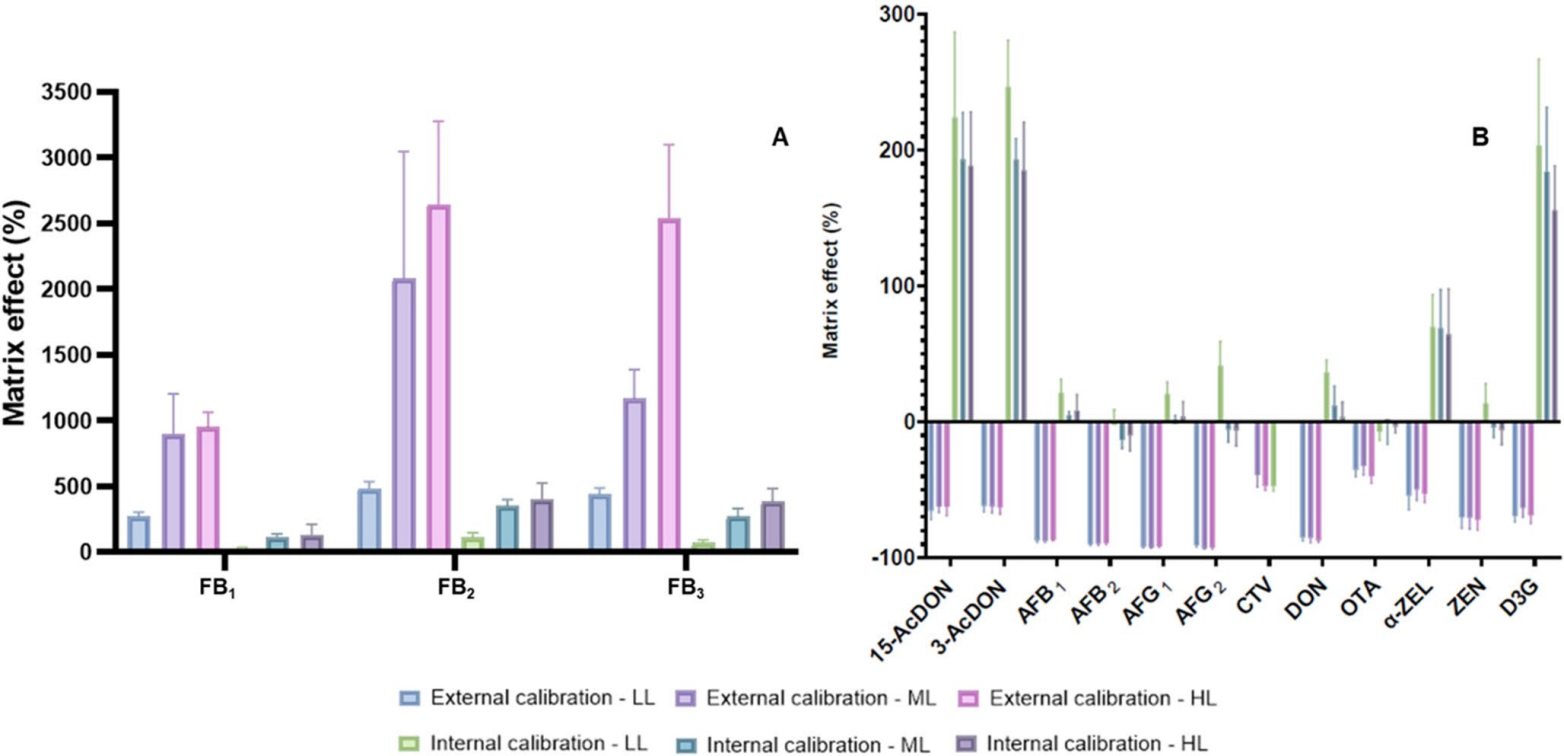
Matrix effects (%) for external and internal calibration at three calibration levels (LL = low level; ML = medium level; HL = high level; *n* = 7–9 replicates at each level), for each mycotoxin evaluated. No internal standard was available for CTV. **A**) Results for fumonisins. **B**) Results for deoxynivalenol and its derivatives, aflatoxins, CTV, ochratoxin A, zearalenone and its derivatives

The chromatogram of the composite blank sample showed no interfering peaks at the same retention times or ion ratios as the analytes of interest, indicating that the method has good selectivity. Matrix effects were evaluated using both external and internal calibration. Using external calibration, only FBs (FB_1_, FB_2_, and FB_3_) showed signal enhancement, ranging from 270% (FB_1_/first level of calibration curve) to 2492% (FB_2_/fifth level of calibration curve; Fig. 2A). Ion suppression was observed for all other analytes, with AFG_2_ showing the highest suppression (mean of -92.3% ± 1.2) and OTA the lowest (mean of -36.1% ± 9.8; Fig. 2B).

Internal calibration using isotope dilution reduced matrix effects for most analytes, keeping signal suppression/enhancement below 20% for 7 out of 14 analytes evaluated (Fig. 2). FBs still showed enhanced signal with internal calibration (70.5–252%), although lower than with external calibration (Fig. 2A). D3G, 15AcDON, and 3AcDON showed higher matrix effects with internal calibration (153 to 202%) than with external calibration (-68 to -63%), and therefore the labeled internal standard d1-DON was not considered suitable for those compounds. Considering the results above, internal calibration was used for all analytes, except D3G, 15-AcDON, and 3-AcDON. An internal standard was not available for CVT. Analytical curves were prepared in-matrix using the composite sample.

Validation results are presented in Table 1. Homoscedastic behavior of the analytical curve residues (F_calc_ < F_crit_) was observed only for 15-AcDON, 3-AcDON and α-ZEL. For heteroscedastic compounds, the best weighting factors were 1/x^2^ (AFB_1_, AFB_2_, AFG_2_, CTV, FB_1_ and ZEN), 1/y^2^ (AFG_1_, D3G and FB_2_), 1/x (DON and FB_3_) and 1/y (OTA). Coefficients of determination were higher than 0.99, except for FB_1_ (0.98); regressions were significant (*p* < 0.05) and did not show any lack-of-fit for all analytes evaluated (data not shown).

**Table 1:** Validation results for the analysis of mycotoxins in a composite matrix, spiked at three different concentration levels

Mycotoxin			Recovery (RSDr).% (*n* = 5–6)			RSDp. % (*n* = 9–12)		
	Weighting factor^a^	LOQ/LOD (µg/kg)	Low	Medium	High	Low	Medium	High
AFB_1_	1/x^2^	4.8/1.6	81.9(5.7)	89.7(8.5)	82.4(3.2)	8.8	10.7	13.2
AFB_2_	1/x^2^	4.8/1.6	97(6)	87.3(6.9)	85(8.5)	14.4	7.3	9.8
AFG_1_	1/y^2^	3.6/1.2	88.1(5.1)	90.2(10.0)	84.1(4.0)	15.0	12.4	12.4
AFG_2_	1/x^2^	4.8/1.6	96.2(8.9)	83.5(3.6)	86.7(9.6)	10.2	9.0	8.8
CTV	1/x^2^	9.6/1.8	95.4(16.0)	114.3(7.8)	115.5(6.8)	14.9	13.4	11.3
DON	1/x	201/67	92.4(9.6)	105.8(11.3)	100.1(8.1)	10.8	11.9	9.2
15-AcDON	Ordinary	90/30	98.4(8.8)	97.8(8.1)	94.8(7.3)	7.0	6.6	7.9
3-AcDON	Ordinary	71/24	93.3(11.4)	90.6(5.7)	89.2(6.0)	8.8	5.9	5.4
D3G	1/y^2^	120/40	95.1(10.9)	83.2(11.2)	102.3(7.6)	10.7	9.6	13.1
FB_1_	1/x^2^	34/11	80.5(5.5)	80.6(8.5)	92.3(12.0)	6.5	14.7	12.5
FB_2_	1/y^2^	2.2/0.7	107.1(6.1)	91.7(10.8)	98.4(8.7)	12.4	10.8	14.3
FB_3_	1/x	2.2/0.7	110.7(19.9)	86.4(9.9)	89.2(13.1)	19	12	13.6
OTA	1/y	2.4/0.8	86.8(13.2)	97.6(17.5)	82.8(10.1)	12.8	12.4	8.3
ZEN	1/x^2^	5.3/1.8	102.1(6.8)	89.5(5.7)	83.8(6.5)	10.6	9.8	12.1
α-ZEL	Ordinary	4.55/1.5	93.1(7.1)	97.3(8.8)	88.9(8.3)	9.5	15.2	10.0

LOQ: limit of quantification; LOD: limit of detection; %RSDr: relative standard deviations obtained from replicates used in the recovery experiments; %RSDp: relative standard deviations obtained from replicates of intermediate precision experiments; ^a^ R^2^ >0.99 for all compounds evaluated, except for FB_1_ (0.98); the regressions were significant (*p* < 0.05) and did not show any lack-of-fit for all analytes

Recoveries ranged from 81.9% (AFB_1_) to 111% (FB_3_) at the lowest level of fortification and from 82.4% (AFB_1_) to 116% (CTV) at the highest level, using matrix-matched curves and an isotope internal standard for quantification, except for 15-AcDON, 3-AcDON, D3G and CTV for which quantification was carried out using only matrix-matched curves. Both RSDr and RSDp were below 20% for all mycotoxins in all three levels evaluated. Limits of quantification (LOQs) ranged from 2.2 µg/kg (FB_2_ and FB_3_) to 201 µg/kg (DON) (80 ≥ recovery ≤ 120%; RSD_r_ ≤ 20%; RSD_P_ ≤ 20%) and LODs from 0.7 µg/kg (FB_2_ and FB_3_) to 67 µg/kg (Table 1).

### Mycotoxin occurrence in dry herbs used for tea preparation

A total of 91 dry herb samples were analyzed using the validated method, of which 35 (38.5%) were positive (≥ LOD) for at least one mycotoxin. Samples with contamination levels above the linear range of the analytical curve were diluted prior to quantification. Twenty-three samples (25.3%) had quantifiable levels (≥ LOQ) of at least one mycotoxin, of which five showed co-occurrence of different mycotoxins (one with AFB_1_ and OTA, one with FB_2_ and ZEN, one with OTA and ZEN and two with tZEN). ZEN was the most frequent quantified mycotoxin (13.2% of the samples), followed by FB_2_ (4.4%), AFB_1_ (3.3%) and OTA (3.3%). Trace levels (LOD ≥ Sample < LOQ) were found in 22 samples, most of which were contaminated with ZEN and FB_2_. Only ten of the 33 different dry herb types did not contain any of the analyzed mycotoxins (< LOD) (*Arnica*,* Assa-peixe*,* Barbatimão*,* Boldo*,* Carqueja*,* Cáscara sagrada*,* Chapéu de couro*, Chlorella, Peruvian maca and Spirulina).

A summary of the results is shown in Table 2, and the results of each sample are in Table S1. Aflatoxins (AFB_1_, AFB_2_, AFG_1_) were quantified in 7 samples at levels ranging from 7.7 (AFG_1_; Muira puama, bark) to 465.2 µg/kg (AFB_2_; Senna, leaf and bark). Three samples contained aflatoxins at trace levels, and there was no co-occurrence among them. FB_2_ was the only fumonisin detected in the samples (11 samples), mainly guarana (3 samples), and seven at trace levels (≥ LOD< LOQ); levels in the four quantified samples ranged from 2.2 (Guarana seed) to 10.1 µg/kg (Angelika leaf). Seven samples were contaminated with OTA, 3 of them at levels between 2.7 (Tribulus, fruit dry extract) and 11.6 µg/kg (Gotu kola, leaf; Figure S4). OTA was also detected in trace levels in samples of Angelika (leaf), Cat’s claw (bark), Guarana (seed) and Horse chestnut (seed).

Nineteen samples were contaminated with ZEN, of which 12 were at levels ≥ LOQ; three horsetail samples contained the highest levels found in the study (from 366.3 to 1954.8 µg/kg; Table 2). α-ZEL, a ZEN metabolite, was found in two horsetail samples, at levels of 7.0 and 39.9 µg/kg. DON and its derivatives (15-AcDON, 3-AcDON and D3G), CTV, fumonisins B_1_ and B_3_ were not found in any of the samples analyzed.

**Table 2:** Occurrence of mycotoxins in dry herb samples used for tea preparation collected in the Federal District, Brazil

Mycotoxin	Positive samples^a^ (%)	Quantified samples (%)^b^	Mean ± SD (Range), µg/kg
AFB_1_	3 (3.3)	3 (3.3)	15. 4 ± 11.5 (7.8–28.6)
AFB_2_	4 (4.4)	2 (2.2)	256.1 ± 295.7 (47.1-465.2)
AFG_1_	2 (2.2)	2 (2.2)	35.1 ± 38.7 (7.7–62.4)
AFG_2_	1 (1.1)	0	–
FB_2_	11 (12.1)	4 (4.4)	5.0 ± 3.5 (2.2–10.1)
OTA	7 (7.7)	3 (3.3)	8.1 ± 4.7 (2.7–11.6)
ZEN	19 (20.9)	12 (13.2)	280.2 ± 585.5(6.5-1954.8)
α-ZEL	3 (3.3)	2 (2.2)	23.4 ± 23.3 (7.0-39.9)

^a^ ≥ LOD; ^b^ ≥ LOQ; SD = standard deviation

### Chronic dietary risk assessment

The assessment was performed for mycotoxins detected in at least 10% of the samples (≥ LOQ and trace levels), namely fumonisin B_2_ and total zearalenone (tZEN = ZEN + α-ZEL) (Table 2). The established PMTDI is 2 µg/kg bw day for fumonisins (FB_1_, FB_2_, FB_3_ – alone or in combination; (JECFA, 2002) and the TDI for ZEN and its metabolites is 0.25 µg/kg bw day (EFSA 2016). Risk may exist when exposure exceeds the PMTDI/TDI.

Results of dietary exposure are shown in Table 3. Estimated FB_2_ intake ranged from 0.0171 ng/kg bw day (mean consumption and median contamination level) to 0.2049 ng/kg bw day (high consumption and P95 of contamination level), representing 0.001% to 0.01% of the fumonisins PMTDI. For tZEN, the intake ranged from 0.0750 to 12.8 ng/kg bw day, representing 0.03% to 5.1% of the TDI. These results indicate that herbal tea consumption is not a major source of exposure to these mycotoxins, even under extreme scenarios.

**Table 3:** Dietary risk assessment of FB2 and tZEN (ZEN + α-ZEL) through the consumption of tea infusions

Mycotoxin	Consumption (g/day)^a^	Contamination (µg/kg)		Intake^b^ (ng/ kg bw day)		% PMTDI/TDI	
		Median	P95	Median	P95	Median	P95
FB_2_	3	0.4	0.7	0.0171	0.0342	0.001	0.002
	18			0.1025	0.2049	0.01	0.01
tZEN	3	1.6	46.3	0.0750	2.1381	0.03	0.9
	18			0.4500	12.8285	0.2	5.1

FB_2_ = fumonisin B_2_; tZEN = total zearalenone; P95 = 95th percentile ; PMTDI = Provisional Maximum Tolerable Daily Intake; FB_2_ = 2 µg/kg bw (JECFA, 2002); TDI = Tolerable Daily Intake; tZEN = 0.25 µg/kg bw (EFSA 2016); ^a^ mean consumers − 1 cup of tea per day (3 g/200 mL) and high consumers – 6 cups/day; ^b^ body weight of 65 kg for the adult population

## Discussion

This study optimized and validated a method for the simultaneous analysis of 15 mycotoxins in 33 different types of dried herbs commonly used for tea preparation. Samples were extracted and cleaned up using a modified QuEChERS approach, as reported in other studies (Caldeirão et al. 2021; Cho et al. 2019; Fontana et al. 2024; Pallarés et al. 2022a, b). All of them used acidified acetonitrile below 2% and a QuEChERS clean-up mixture containing C18, except for Fontana et al. (2024), who used graphitized carbon black (CGB). In the present study, FBs and OTA were better extracted with 10% acidified acetonitrile, which was used in the method. NaCl was used as a dispersion salt, and PSA and C18 were used in the cleanup step. PSA was needed to improve the recovery of AFB_1_, AFG_1_, CTV, 15-AcDON, 3-AcDON and DON when compared with using only C18. The analytical method was validated using a composite sample containing different herbs and plant parts. Matrix effects were higher than previously reported in the literature, mainly for fumonisins (up to 2,000% using external calibration). For example, a method that also used QuEChERs prior to UHPLC-MS/MS reported matrix effects values of -1 to 51% for FB_1_ and − 16 to -31% for FB_2_, depending on the dry herb (Fontana et al. 2024). The study also reported matrix effects for aflatoxins ranging from − 12% (AFG_2_) to -71% (AFB_2_) for *Melissa officinalis* and from − 20% (AFB_1_) to 31% (AFG_2_) for *Malva sylvestris*. The present study was the only one to report the use of a composite sample for validation procedures. Other researchers analyzed a limited number of plant species (Fontana et al. 2024; Pallarés et al. 2022a, b), validated the method using individual species (Zhou et al. 2022b), or used different species to represent different plant parts (Cho et al. 2019). To the best of our knowledge, this is the first study to report the use of isotope dilution for the multi-mycotoxin analysis in dry herbs used for tea preparation. Except for fumonisins, signal suppression was observed with external calibration, whereas signal enhancement was observed with internal calibration, especially for DON and its derivative/metabolites.

The LOQs established for the present method were within the same range as those reported by Fontana et al. (2024) for aflatoxins (5 µg/kg), DON (250 µg/kg- *Melissa officinalis*), but were considerably lower for fumonisins (FB_1_ and FB_2_, 500 µg/kg), OTA (10 µg/kg) and ZEN (250–500 µg/kg). Reinholds et al. (2019) achieved lower LOQs for AFB_1_ (0.4 µg/kg), OTA (0.8 µg/kg) and DON (34 µg/kg) using modified QuEChERS, followed by HPLC-TOF-MS; however, they developed a separate method for DON analysis. Lower LOQs were also achieved by Wan et al. (2025) for DON (8.3 µg/kg), 3-AcDON (5.0 µg/kg), 15-AcDON (6.0 µg/kg), AFs (0.7–0.8 µg/kg) and OTA (0.7 µg/kg), although the present method obtained better results for FB_1_/FB_2_ (82.5 µg/kg) and ZEN (7.5 µg/kg). Wan et al. (2025) used only fresh leaves of *Camellia formosensis* for the validation procedures. The method’s low sensitivity to DON and its derivatives may have contributed to the failure to detect them in some samples in the present work.

About 25% (*n* = 23) of the analyzed samples were contaminated with at least one mycotoxin and co-occurrence of two or more mycotoxins was found in 5% of the samples (*n* = 5). ZEN and FB_2_ were the main mycotoxins found. Samples of horse chestnut, green tea, senna, and horseradish showed the highest aflatoxin levels, and those of mulungu and horsetail contained the highest ZEN levels. Stevic et al. (2012) evaluated microbiological characteristics of several medicinal plants and found that horsetail was one of the most contaminated with molds, mainly from *Fusarium* and *Aspergillus*. Since some *Fusarium* species produce ZEN, the high levels of contamination found in horsetail samples may be related to the affinity of these fungi for the plant.

Table 4 summarizes published studies on the occurrence of mycotoxins in dry herbs used for tea preparation. Two studies evaluated samples collected in Brazil. Caldeirão et al. (2021) analyzed 58 samples from 20 different dry herbs, and found samples contaminated with aflatoxins (7–17% of the samples) and OTA (19%); both occurrence and contamination levels were higher than those found in the present study, except for AFB_2_ (up to 465.2 µg/kg; Table 2). Fontana et al. (2024) did not detect any of the mycotoxins investigated in the 42 analyzed samples, and the authors reported that appropriate drying and storage procedures were used for the products. However, it is important to emphasize that fungus infection and mycotoxin production may start in the field (Taniwaki et al. 2018; Zhang et al. 2022), and although adequate drying and storage may control aflatoxin contamination (Müller and Basedow 2007; Pitt et al. 2013), it does not eliminate mycotoxins already present in the herbs.

**Table 4:** Published studies on the occurrence and exposure assessment of mycotoxins in dry herbs used for tea preparation, as reported in each publication

Country / *n*° mycotoxin analyzed (reference)	Type of tea (*n*° samples)	Mean contamination level (range), µg/kg	Mean Intake, ng/ kg bw day (%PMTDI/HQ/MOE/Risk of cancer)
Brazil / 14 (Caldeirão et al. 2021)	20 different species (58)	AFB_1_: NR (74–1,993) AFB_2_: NR (49–184) AFG_1_: NR (99–1,627) OTA: NR (45–404)	AFs: 0.009–0.022 (18–44)^a^ OTA: 0.018 (805)^b^
Brazil / 11 (Fontana et al. 2024)	*Melissa officinalis* and *Malva sylvestris*m (42)	AFs, DON, FBs, OTA and ZEN: ND	NE
China / 7 (Chen et al. 2020)	13 different species (48)	AFB_1_: NR (0.1–3.8) AFB_2_: NR (0.4–0.5) AFG_1_: NR (0.8) AFG_2_: NR (0.9–2.1) OTA: NR (0.3–515)	NE
China / 16 (Zhou et al. 2022a)	Green, oolong, black, and dark tea (352)	AFs: 1.3–10.6 (NR – 47.2) OTA: 0.09–129.8 (NR – 11,354) ZEN: 0.17–2.1 (NR – 8.4) α-ZEL: 17.8–24.3 (NR – 116.2) DON: 15.9 − 184 (NR – 1,748) 3-AcDON: 7.1–7.8 (NR – 36.6) 15-AcDON: 15–84.7 (NR – 748)	AFs: NR (0.00118–0.499)^c^ OTA: 0.003–7.2 (0.00021–0.51)^d^ TZEN: 3.4–7.7 (0.014–0.031)^d^ TDON: 0.921–113 (0.0009–0.113)^d^
Korea / 11 (Cho et al. 2019)	20 different species (100)	AFB_1_: 5 AFB_2_, AFG_1_, AFG_2_: ND OTA: 25.3 (1.4–58.3) ZEN: 7.4 (2.9–15.2) DON: 28.9 (2.1 – 128.9) FB_1_: 17.2 (0.8–33.7) FB_2_: 1.3 (0.9–1.6) FB_3_: 47.2 (0.7–205.4)	NE
Latvia / 42 (Reinholds et al. 2020)^e^	Camellia sinensis (140) and herbal teas (26)	AFs: 1.5–8.2 (1.4–103) OTA: 0.6–3.0 (1.1–7.7) TDON: 103–3,599 (97.1–17,360) ZEN: 11.3 (NR – 56.1)^f^	AFs: 0.0764–0.2933 (0.04–0.17)^g^ OTA: 0.0296–0.11 (0.17–2.05)^h^ TDON: 4.7–129 (1.0–78.9)^h^ ZEN: 0.513 (0.20–1.1)^f, h^
Morocco / 15 (Jai et al. 2021)	Green tea (111)	AFB_2_: 0.13 (4.9–7.4) AFG_1_: 0.03 (1.1–1.6) ZEN: 2.6–9.4 (15.3–45.8)	AFB_2_: 0.01–1.8 (NE)^i^ AFG_1_: 0.002–0.3 (NE)^i^ ZEN: 0.6–1.3 (0.2–0.5)^i^
Portugal /38 (Assunção et al. 2021)	Green tea, bulk and bags (20)	NR	AFB_1_: 0.00002–0,00028 (17,445,715–1,428,571)^j^ ZEN: 0.0003–0.0036 (0.000005–0.000014)^k^ FB_1_: 0.0005–0.0063 (0.000005–0.000063)^k^
Portugal / 5 (Duarte et al. 2020)	Tea and medicinal plants (37)	AFs: 14.7 (2.8 − 8.2) ZEN: 8.9 (1.8–19.0)	AFB_1_: 0.02–0.24 (12.1–122.5)^l^ ZEN: 0.02–0.17 (0.01 − 0.07)^l^
Taiwan / 16 (Wan et al. 2025)	Green, oolong, black, and Pu-erh (8)	AFM_1_: 2.6 (2,15–3.0) FB_2_: 198.9 ZEN: 87.5	AFM_1_: 0.09–0.3 (46,200–9,561)^m^ FB_2_: 11.1–39.1 (0.5–2.0)^m^ ZEN: 1.4–4.9 (0.6–2.0)^m^

PMTDI = provisional maximum tolerable inclusion of daily before intake; HQ = hazard quotient; MOE: Margin of exposure (each parameter was reported according to the mycotoxin evaluated and the study methodology); NR: not reported; ND: not detected (samples < LOD); NE = not estimated; tZEN = ZEN + metabolites; tDON = DON + metabolites; ^a^Estimation made for every kind of dry herbs; MOE estimated considering a BMDL10 of 0.4 µg/kg bw day; ^b^Estimation made for every kind of dry herbs; MOE estimated considering a BMDL10 of 14.5 µg/kg bw day; ^c^Reported as cancer risk (cancer/year 10^5^ individuals); ^d^Reported as HQ (HQ = Intake/PMTDI) - DON and its acetylated derivatives = 1.0 µg/kg bw/day, ZEN and its modified forms = 0.25 µg/kg bw/day and OTA = 0.0143 µg/kg bw/day; ^e^ Estimation made both for upper bound and maximum concentration levels; ^f^only for Pu-erh samples; ^g^MOE estimated considering a BMDL10 of 170 ng/kg bw day; Values described were not consistent with the MOE estimation; ^h^DON and its acetylated derivatives = 1.0 µg/kg bw/day, ZEN and its modified forms = 0.25 µg/kg bw/day and OTA = 17.1 ng/kg bw/day; ^i^Lower – upper bound; TDI (ZEN) = 0.25 µg/kg bw/day; ^j^Just one contaminated sample; MOE estimated considering a BMDL10 of 0.4 µg/kg bw day; ^k^Just one contaminated sample; Reported as HQ (HQ = Intake/TDI); TDI (ZEN) = 0.25 µg/kg bw/day; FB_1_ = 0.1 µg/kg bw/day; ^l^TDI (AFs) = 0.2 ng/kg bw/day; TDI (ZEN) = 0.25 ng/kg bw/day; ^m^Exposure was estimated for two age groups (19–65 years old and > 65 years old) and two populational groups (whole and consumers only); For AFM_1_, MOE was estimated considering a BMDL10 of 0.4 µg/kg bw day; TDI (ZEN) = 0.25 µg/kg bw/day; TDI (FB_1_/FB_2_) = FB_1_ = 2 µg/kg bw/day

The types of herbs analyzed, as well as the occurrence and levels of mycotoxins found, vary widely across studies conducted elsewhere (Table 4). In a large study conducted in China (352 samples), OTA (11,354 µg/kg in dark tea) and DON (1,748 µg/kg, oolong) were present at the highest levels (Zhou et al. 2022a). Cho et al. (2019) found up to 10% of the 100 functional and medicinal herb Korean samples contaminated, with the highest levels for DON (128.9 µg/kg) and FB_3_ (205.4 µg/kg). Reinholds et al. (2021) found that about 40% of the 166 samples from Latvia were contaminated with aflatoxins, 66% with DON and its derivatives, with a tDON level (DON, D3G, 3-AcDON, 15-AcDON) reaching 17,360 in a Pu-erh sample. Jai et al. (2021) found 2% of the 111 samples from Morocco contaminated with AFB_2_ and/or AFG_1_, and 35% with ZEN (up to 45.8 µg/kg). Duarte et al. (2020) detected AFs and ZEN in tea and medicinal plants from Portugal and Wan et al. (2025) found FB_2_ in one green tea sample and ZEN in one black tea sample.

In Brazil, herbal teas are regulated either as food or as herbal medicines, which may claim medicinal use (ANVISA, 2022); however, medicinal plants can also be used for tea preparation. From the 91 herb samples analyzed, nine fell under the food category, nine as medicinal plants, 11 could be used in the preparation of herbal medicine, 32 belonged to two categories (e.g. food and herbal medicine/medicinal plant and herbal medicine) and 30 samples were not listed in any category in the legislation, as they were composed of different plant species than described in the technical documents, other plant parts or not described at all. Brazilian MLs are established only for dry herbs used as medicinal plants (AFs = 20 µg/kg; AFB_1_ = 5 µg/kg). If these limits were applied to all types of herbs analyzed in the present study, samples of green tea (leaf and stalk; AFB_2_ = 47.1 µg/kg), horse chestnut (seed; AFB_1_=28.6 µg/kg), horseradish tree (leaf; AFG_1_=62.4 µg/kg) and sena (leaf and bark; AFB_2_ = 465.2 µg/kg) would exceed the MLs.

The Brazilian Health Surveillance Agency requires producers to monitor mycotoxins in dry herbs used for herbal medicine preparation whenever reports on their occurrence are available (ANVISA, 2019). However, to our knowledge, no published compilation of this information exists, making it challenging to identify which plants and mycotoxins should be monitored in dry herbs in Brazil. The present study identified eight mycotoxins in at least 22 species of dry herbs, information that could be used to build a database to support producer monitoring actions. Furthermore, discussions on the establishment of MLs for mycotoxins in herbal teas commercialized in Brazil should be undertaken, taking into account both the present study and available literature. 

The chronic dietary risk assessment for tZEN and FB2 showed no risk of exposure from tea infusion consumption among the Brazilian population. As a conservative approach, this study considered that all mycotoxins present in the dry herb were transferred to the infusion ready for consumption, an assumption supported by Reinholds et al. (2019) for ZEN. Other studies, however, showed decreases in mycotoxin concentrations in the herb infusion, depending on the mycotoxin and the infusion preparation. Caldeirão et al. (2021) showed that no AFG_1_ and OTA were transferred to the infusion, and that a reduction of up to 95% in AFB1 content could be achieved during tea preparation, considering the physical-chemical properties of the compounds. In a study by Chalyy et al. (2021), 83% of the initial OTA content was transferred to the infusion, whereas ZEN showed a 75% to 100% reduction. Wan et al. (2025) evaluated naturally contaminated tea samples for ZEN, FB_2_ and AFM_1_ and found that less than 1% of the mycotoxin levels were transferred into tea infusion during the first brewing (up to 2% after five brews). If a decrease in mycotoxin concentration were considered in the present study, the estimated exposure would be even lower, as would the risks from tea consumption.

Other studies also showed that the risk of mycotoxins from tea consumption was low, even when conservative approaches were used (Table 4). In Brazil, Caldeirão et al. (2021) evaluated risks from exposure to aflatoxins (AFB_1_ and AFB_2_), OTA, sterigmatocystin and HT-2 (a trichothecene) considering daily tea consumption of 200 mL of individual types of herbs (rosemary, star anise and sage). The hazard quotient (HQ) calculated for HT-2 showed a potential health concern in infusions of *espinheira santa* (4.5) and mint (2.2). The margin of exposure (MOE) for aflatoxins was below 50 (Table 4) for rosemary, star anise and sage, which may indicate a potential health risk for a genotoxic compound, since an MOE below 10,000 is of concern. MOE was 805.5 for OTA and 26.7 for STC, but as these mycotoxins are not genotoxic, no potential health risk is expected.

Using both deterministic and probabilistic approaches, Zhou et al. (2022a) found no potential risk to the Chinese population from the exposure to 16 mycotoxins through the consumption of tea as a beverage or a dietary supplement (green, oolong, black and dark tea). Reinholds et al. (2020) estimated that the exposure of the Latvian population to mycotoxins through the consumption of tea (black, green, oolong and Pu-erh) represented up to 78.9% of the TDI for DON. Although the authors stated that there were no health risks for AFs, the MOE estimated from the data presented in the paper was < 10,000, indicating a health concern.

Assunção et al.(2021) estimated intake of AFB1, FB1, and ZEN from green tea consumption in Portugal (10 cups, 150 mL each) and found no potential health risks. However, exposure was estimated based on only one positive sample per mycotoxin (≥ LOD), which is very limited. In another Portuguese study, exposure to AFs and ZEN through tea consumption ranged from 12.1 to 122% of the Tolerable Daily Intake (TDI) for AFs and up to 0.07% of the TDI for ZEN (Duarte et al. 2020). However, the TDI approach for AFs is not recommended as they are genotoxic compounds. In Taiwan, mycotoxin exposure from tea brewed up to 5 times (consumers only) and individual concentrations in contaminated samples reached up to 2% of the established TDI for ZEN and FB_2_ (Wan et al. 2025).

Although most studies showed no health concerns from mycotoxin exposure through tea consumption, some have indicated a potential health risk from aflatoxin exposure based on a very small number of positive samples. In the present study, the chronic dietary risk assessment was conducted only for FB_2_ and tZEN, due to the low number of positive samples (≥ LOD) for the other mycotoxins, which limited the scope of the assessment.

One limitation of this study is the high LOD/LOQ obtained for DON and its derivatives, which may have affected the ability to detect these mycotoxins in the analyzed samples. It is well established that good agricultural and manufacturing practices contribute to the control of mycotoxin contamination. Therefore, improvements must be implemented throughout the herbal tea production chain to ensure the lowest possible levels of mycotoxin contamination in the final products. Furthermore, continuous monitoring is needed to generate additional occurrence data and support a broader chronic dietary risk assessment.

In summary, this study established a QuEChERS-based UHPLC-MS/MS method that was fully validated for the simultaneous determination of 15 mycotoxins in complex herbal matrices used for tea preparation. By incorporating composite-sample validation and isotope-labeled internal standards, matrix effects were markedly reduced and quantitative accuracy improved, enabling reliable multi-analyte monitoring of dry herbs. Among the 91 dry herbs analyzed, 25.3% contained at least one mycotoxin and co-occurrence was observed in five samples. Dietary exposure to tZEN and FB_2_ from tea infusions was below health-based guidance values; however, the occurrence of elevated contamination in some samples underscores the need for targeted monitoring and reinforced producer-level control measures.

## Electronic Supplementary Material

Below is the link to the electronic supplementary material.

Supplementary Material 1

## Data Availability

The datasets generated and/or analyzed during the current study are available from the corresponding author upon request.
