# Supplementary material for: Mycotoxins occurrence in dry herbs used for tea preparation: method validation, analysis of bulk samples and dietary risk assessment
Source: Mycotoxin Res. 2026 May 23;42(3):41. doi: 10.1007/s12550-026-00652-2 (PMC13198502; doi:10.1007/s12550-026-00652-2)
Supplement: Supplementary file 1 — Supplementary Material 1 [file 12550_2026_652_MOESM1_ESM.docx]

**SUPPLEMENTARY MATERIAL**

**Mycotoxins occurrence in dry herbs used for tea preparation: method validation, analysis of bulk samples and dietary risk assessment**

**Camila Suguiura Evangelista^1^, Denise Carvalho Mello^1^, Eloisa Dutra Caldas^1^, Patrícia Diniz Andrade^1,2^**

^1^Laboratory of Toxicology, Faculty of Health Sciences, University of Brasília, Brasilia, DF 70910-900, Brazil

^2^Faculty of Agronomy and Veterinary Medicine, University of Brasília, DF 70910-900, Brazil

**Table S1.** Number of samples collected in the Federal District between 2018 and 2021.

| **Sample (scientific name)** | **Plant part** | **Manufacture's recommendation (g)** | **N** | **Positive samples** | **Mycotoxin** | **Level, µg/kg** |
| --- | --- | --- | --- | --- | --- | --- |
| Angelica (*Angelica officinalis L.*) | Leaf | 2 - 20 | 2 | 1 | FB_2_ | 10.1 |
|  |  |  |  |  | OTA | Traces (≥0.8 <2.4) |
|  |  |  |  |  | ZEN | Traces (≥1.8 <5.3 |
| Arnica (*Arnica montana*) | Bark and leaf | 3 | 1 | < LOD | - | - |
| Arnica-do-mato (*Solidago microglossa*) | Leaf | 3 | 1 | < LOD | - | - |
| Artichoke (*Cynara scolymus*) | Leaf | 3 - 10 | 5 | 1 | FB_2_ | 2.9 |
| Assa-peixe (*Vernonia polysphaera*) | Leaf | 3 | 1 | < LOD | - | - |
| Barbatimão (*Stryphnodedron barbatiman*) | Bark | 3 | 1 | < LOD | - | - |
| Black mulberry (*Morus nigra*) | Leaf | 3 - 10 | 2 | 1 | ZEN | 52.0 |
|  |  |  |  |  | α-ZEL | Traces (≥ 1.5 <4.5) |
| Bladder wrack (*Fucus vesiculosus*) | Leaf | 9 - 12 | 3 | 1 | AFB_2_ | Traces (≥ 1.6 <4.8) |
| Boldo (*Boldus boldus*) | Leaf | 3 - 10 | 1 | < LOD | - | - |
| Boldo (Peumus boldus) | Leaf | 3 - 10 | 6 | < LOD | - | - |
| Canela de velho (*Miconia albicans*) | Leaf | NR | 2 | 1 | ZEN | Traces (≥ 1.8<5.3) |
| Carqueja (*Baccharis trimera*) | Leaf | 10 | 1 | < LOD | - | - |
| Cáscara sagrada (*Rhamnus purshiana*) | Bark | 3 | 2 | < LOD | - | - |
| Cat’s claw. (*Uncaria tomentosa*) | Bark | 3 | 6 | 2 | AFB_1_ | 9.8 |
|  |  |  |  |  | AFB_2_ | Traces (≥ 1.6 <4.8) |
|  |  |  |  |  | FB_2_ | Traces (≥1.6 <4.8) |
|  |  |  |  |  | OTA | Traces (≥ 0.8 < 2.4); 10.0 |
| Chamomile (*Matricaria chamomilla*) | Flower | 3 - 10 | 1 | < LOD | - | - |
| Chamomile (*Matricaria recutita*) | Flower | 3 - 10 | 1 | 1 | ZEN | 6.6 |
| Chamomile (*Matricaria chamomilla*) | Flower and aerial part | 3 - 10 | 1 | < LOD | - | - |
| Chamomile (*Matricaria recutita*) | Flower, leaf and stem | 3 - 10 | 1 | < LOD | - | - |
| Chamomille (Matricaria recutita) | Leaf | 3 - 10 | 2 | 1 | ZEN | Traces (≥ 1.8 <5.3) |
| Chapéu de couro (*Echinodorus macrophyllus*) | Leaf | 3 | 3 | < LOD | - | - |
| Chlorella (*Chlorella pyrenoidosa*) | Algae | NR | 2 | < LOD | - | - |
| Comfrey (*Symphytum officinale*) | Leaf | 9 | 1 | 1 | ZEN | 8.3 |
| Espinheira-santa (*Maytenus ilicifolia*) | Leaf | 10 | 5 | 1 | AFB_1_ | 7.8 |
| Ginkgo (*Ginkgo biloba*) | Leaf | 3 | 2 | 1 | FB_2_ | 4.7 |
|  |  |  |  |  | ZEN | 6.5 |
| Gotu kola (*Hydrocotyle asiatica*) | Leaf | 3 | 2 | 1 | FB_2_ | Traces (≥ 0.7 < 2.2) |
|  |  |  |  |  | OTA | 11.6 |
|  |  |  |  |  | ZEN | 9.2 |
| Green tea (*Camelia sinensis*) | Leaf | 3 - 18 | 3 | 2 | AFG_2_ | 3.2 |
|  |  |  |  |  | ZEN | Traces (≥ 1.8 < 5.3) |
| Green tea (*Camelia sinensis*) | Leaf and stalk | 3 - 18 | 1 | 1 | AFB_2_ | 47.1 |
| Guarana (*Paullinia cupana*) | Seed | 3 | 3 | 3 | FB_2_ | Traces (≥ 0.7 < 2.2); 2.2 |
|  |  |  |  |  | OTA | Traces (≥ 0.8 < 2.4) |
| Hibiscus (*Hibiscus sabdariffa*) | Flower | 10 | 1 | 1 | FB_2_ | Traces (≥ 0.7 < 2.2) |
| Hibiscus (*Rosa sinensis*) | Flower | 10 | 1 | < LOD | - | - |
| Horse chestnut (*Aesculus hippocastanum*) | Seed | 3 | 1 | 1 | AFB_1_ | 28.6 |
|  |  |  |  |  | FB_2_ | Traces (≥ 0.7 < 2.2) |
|  |  |  |  |  | OTA | Traces (≥ 0.8 < 2.4) |
|  |  |  |  |  | ZEN | Traces (≥ 1.8 < 5.3) |
| Horsetail (*Equisetum arvense L*) | Aerial part | 3 - 10 | 2 | 2 | ZEN | 366.3;1955 |
|  |  |  |  |  | α-ZEL | 40.0 |
| Horsetail (*Equisetum hyemale*) | Leaf | 3 - 10 | 2 | 1 | ZEN | 867.2 |
|  |  |  |  |  | α-ZEL | 7.0 |
| Horseradish tree (*Moringa oleifera*) | Leaf | 0.1 | 1 | 1 | AFG_1_ | 62.4 |
| Muira puama (*Ptychopetalum olacoides*) | Bark | 3 | 2 | 1 | AFG_1_ | 7.7 |
| Mulungu (*Erythrina velutina*) | Bark | 10 | 2 | 2 | ZEN | 13.6;49.0 |
| Myrcia (*Myrcia multiflora*) | Leaf | 9 - 12 | 1 | < LOD | - | - |
| Passion fruit (*Passiflora alata*) | Leaf | 3 | 1 | 1 | ZEN | 14.5 |
| Passion fruit (*Passiflora incarnata*) | Leaf and stem | 3 | 1 | 1 | ZEN | 14.5 |
| Peruvian maca (*Lepidium meyenii*) | Root | NR | 1 | < LOD | - | - |
| Senna (*Cassia acutifolia*) | Leaf | 3 - 10 | 3 | < LOD | - | - |
| Senna (*Cassia acutifolia*) | Leaf and bark | 3 - 10 | 1 | 1 | AFB_2_ | 465.2 |
| Senna (*Senna alexandrina*) | Leaf | 3 - 10 | 3 | < LOD | - | - |
| Senna (*Cassia angustifolia*) | Leaf | 3 - 10 | 3 | < LOD | - | - |
| Spirulina (*Spirulina plantesis*) | Algae | NR | 2 | < LOD | - | - |
| Tribulus (*Tribulus terrestris*) | Fruit dry extract | NR | 1 | 1 | FB_2_ | Traces (≥ 0.7 < 2.2) |
|  |  |  |  |  | OTA | 2.7 |

N= Number of analyzed samples; SD: standard deviation; LOD: limit of detection;Traces (Tr): ≥ LOD <LOQ; NR: not reported

**Table S2.** Extraction conditions were tested based on modified QuEChERS procedures.

| **Test** | **Water, mL/**  **time, min** | **ACN, mL (% formic acid)** | **Dispersion salt** | | **Clean-up volume (mL)^a^** | **Clean-up** |
| --- | --- | --- | --- | --- | --- | --- |
| 1 | 6.5/30 | 7.5 (1) | 3.75g MgSO_4_+ NaOAc (4:1) | 3 | | 0.6g MgSO_4_+PSA (3:1) |
| 2 | 15/15 | 15 (10) | 5g MgSO_4_+ NaCl (4:1) | 3 | | 0,6g MgSO_4_+C18 (3:1) |
| 3 | 15/15 | 15 (10) | 5g MgSO_4_+ NaCl (4:1) | 3 | | 0.6g MgSO_4_+PSA+C18 (6:1:1) |
| 4 | 15/30 | 15 (10) | 5g MgSO_4_+ NaCl (4:1) | 9 | | 1.26g MgSO_4_+PSA+CGB (3:1) |

^a^Aliquot of the extract used for clean-up.

**Table S3.** Concentration levels (µg/kg) used in the validation procedure for each mycotoxin.

| **Mycotoxin** | **Concentration levels (µg/kg)** | | |
| --- | --- | --- | --- |
|  | **Low** | **Medium** | **High** |
| 15-AcDON | 89.6 | 239.8 | 447.9 |
| 3-AcDON | 71.2 | 190.7 | 356.1 |
| AFB_1_ | 4.8 | 83.2 | 224.6 |
| AFB_2_ | 4.8 | 68.2 | 169.6 |
| AFG_1_ | 3.6 | 68.0 | 170.0 |
| AFG_2_ | 4.8 | 91.6 | 225.3 |
| CTV | 9.6 | 112.9 | 282.2 |
| DON | 200.7 | 537.2 | 1003.3 |
| D3G | 120.2 | 318.6 | 601.2 |
| FB_1_ | 34.3 | 137.2 | 336.1 |
| FB_2_ | 2.2 | 25.0 | 31.5 |
| FB_3_ | 2.2 | 25.0 | 31.5 |
| OTA | 2.4 | 41.2 | 231.6 |
| ZEN | 5.3 | 89.8 | 466.5 |
| α-ZEL | 4.5 | 12.0 | 22.3 |

**Table S4.** Optimized ESI-MS/MS parameters, chromatographic retention times, ion ratios, and internal standards (IS) used for the multi-mycotoxin UHPLC–MS/MS analysis of dry herbs.

| **Analyte** | **DP (V)** | **Transition (m/z)** | **CE (V)** | **CXP (V)** | **RT (min)** | **Ion ratio (RSD; %)** |
| --- | --- | --- | --- | --- | --- | --- |
| 15-AcDON | 106 | 339.1 → 261.1 | 15 | 14 | 6.6 | 0.6 (8.5) |
|  |  | 339.1 → 137.1 | 15 | 16 |  |  |
| 3-AcDON | 96 | 339.0 → 231.1 | 15 | 12 | 6.3 | 0.5 (14.7) |
|  |  | 339.0 → 203.0 | 21 | 12 |  |  |
| U-AFB_1_ (IS) | 126 | 330.1→ 301.0 | 33 | 26 | 11.0 | 0.9 (2.0) |
|  |  | 330.1 → 255.0 | 51 | 28 |  |  |
| AFB_1_ | 121 | 313.0 → 241.0 | 49 | 26 | 11.1 | 1.2 (4.0) |
|  |  | 313.0 → 285.0 | 31 | 14 |  |  |
| AFB_2_ | 136 | 315.0 → 259.0 | 39 | 22 | 10.6 | 0.5 (7.1) |
|  |  | 315.0 → 243.0 | 51 | 26 |  |  |
| U-AFG_1_ (IS) | 16 | 346.1 → 257.1 | 37 | 30 | 10.2 | 0.7 (2.9) |
|  |  | 346.1 → 212.0 | 55 | 24 |  |  |
| AFG_1_ | 101 | 329.0 → 200.0 | 53 | 22 | 10.2 | 1.5 (5.2) |
|  |  | 329.0 → 243.0 | 37 | 28 |  |  |
| AFG_2_ | 136 | 331.0 → 245.0 | 41 | 12 | 9.8 | 1.0 (15.2) |
|  |  | 331.0 → 189.0 | 55 | 20 |  |  |
| CTV | 71 | 403.1 → 297.1 | 21 | 16 | 16.5 | 1.0 (11.6) |
|  |  | 403.1 → 139.0 | 33 | 10 |  |  |
| d1-DON (IS) | 41 | 298.1 → 249.1 | 15 | 14 | 3.0 | 0.4 (5.1) |
|  |  | 298.1 → 203.1 | 21 | 24 |  |  |
| DON | 76 | 297.1 → 249.0 | 16 | 11 | 3.0 | 0.8 (12.2) |
|  |  | 297.1 → 203.2 | 21 | 10 |  |  |
| D3G | -45 | 503.1 → 427.1 | -26 | -23 | 3.2 | 0.4 (15.2) |
|  |  | 503.1 → 247.0 | -28 | -19 |  |  |
| U-FB_1_ (IS) | 36 | 756.5 → 356.2 | 55 | 20 | 15.5 | 0.9 (4.0) |
|  |  | 756.5 → 374.2 | 51 | 18 |  |  |
| FB_1_ | 151 | 723.2 → 352.2 | 49 | 18 | 15.45 | 0.6 (7.7) |
|  |  | 723.2 → 547.2 | 39 | 26 |  |  |
| FB_2_ | 141 | 706.3 → 336.3 | 49 | 18 | 18.2 | 0.5 (8.4) |
|  |  | 706.3 → 318.2 | 51 | 14 |  |  |
| FB_3_ | 161 | 706.3 → 336.2 | 51 | 16 | 16.9 | 0.5 (1.8) |
|  |  | 706.3 → 318.3 | 51 | 16 |  |  |
| U-OTA (IS) | 51 | 424.1 → 249.9 | 33 | 28 | 16.1 | 0.5 (3.5) |
|  |  | 424.1 → 232.0 | 49 | 26 |  |  |
| OTA | 91 | 404.0 → 239.0 | 33 | 12 | 16.1 | 0.3 (4.8) |
|  |  | 404.0 → 102.0 | 85 | 12 |  |  |
| U-ZEN (IS) | -80 | 335.1 → 185.0 | -34 | -9 | 15.7 | 4.1 (19.5) |
|  |  | 335.1 → 139.9 | -40 | -11 |  |  |
| ZEN | -130 | 317.0 → 175.2 | -32 | -15 | 15.7 | 0.8 (5.2) |
|  |  | 317.0 → 130.8 | -36 | -17 |  |  |
| α-ZEL | -130 | 319.0 → 160.0 | -40 | -17 | 15.4 | 0.8 (9.8) |
|  |  | 319.0 → 130.0 | -44 | -15 |  |  |

DP = declustering potential; CE = collision energy; CXP = collision cell exit potential; RT = retention time of the analyte in the chromatographic run; Ion ratio = qualifier/quantifier obtained through the validation experiments; RSD = relative standard deviation; IS = internal standard.


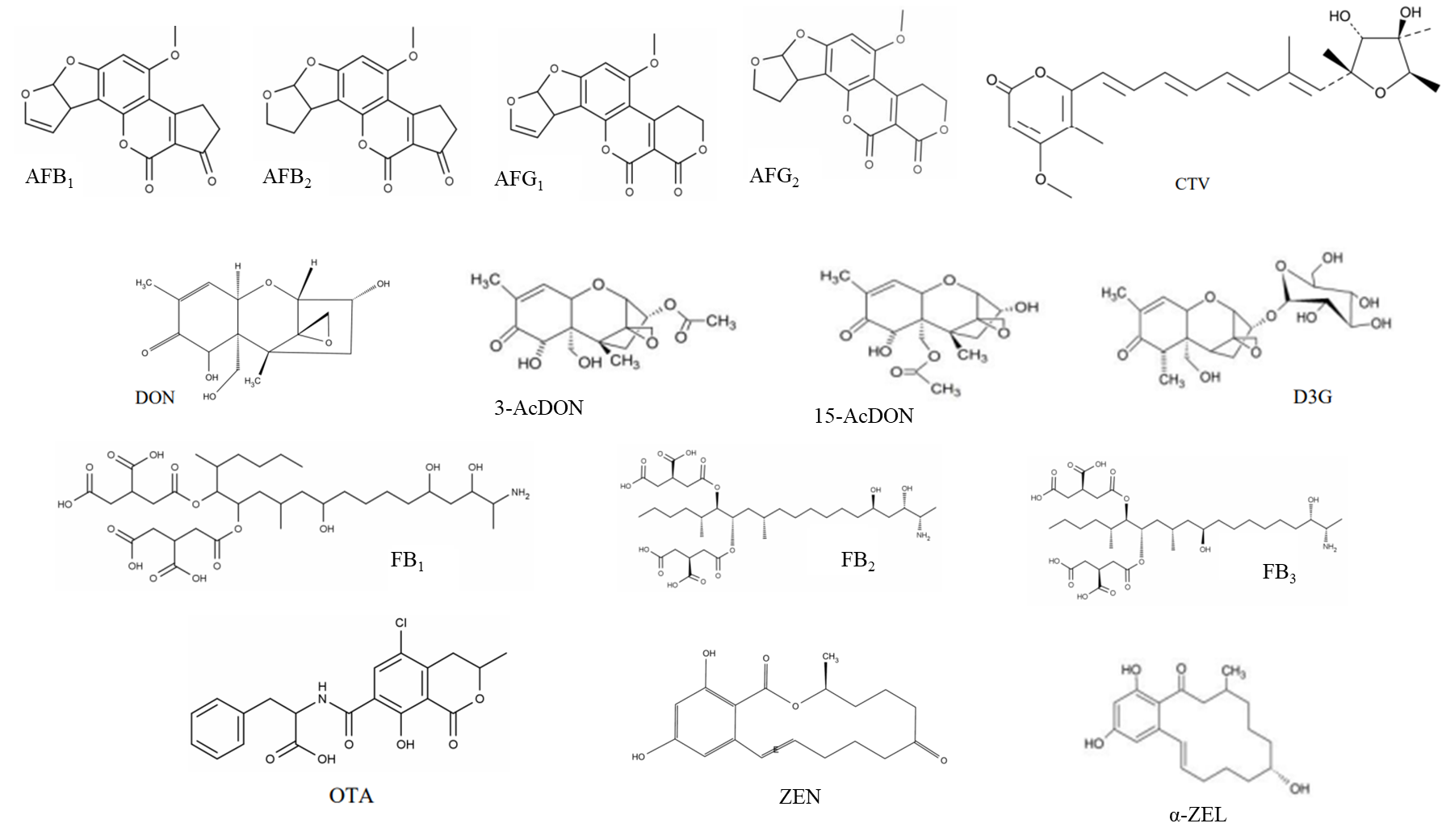


**Figure S1** – Chemical structures of mycotoxins analyzed in the present study


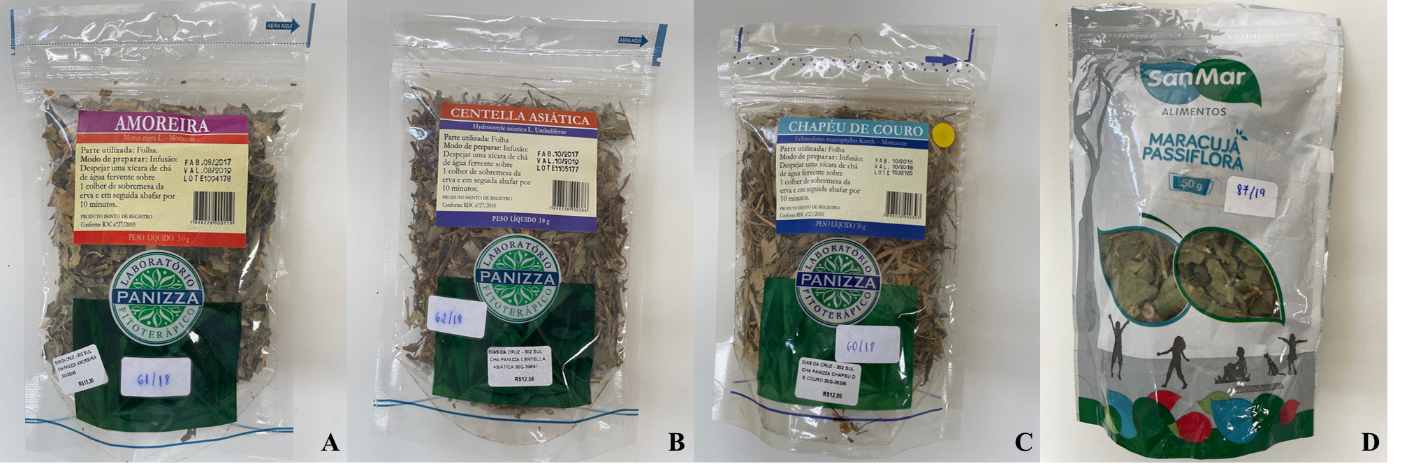
**Figure S2 –** Examples of dry herbs bulk samples purchased from retail stores and compounding pharmacies in the Federal District, Brazil. **A)** Black mulberry (*Morus nigra*) **B)** Gotu kola (*Hydrocotyle asiatica*) **C)** Chapéu de couro (*Echinodorus macrophyllus*) **D)** Passion fruit (*Passiflora alata*).


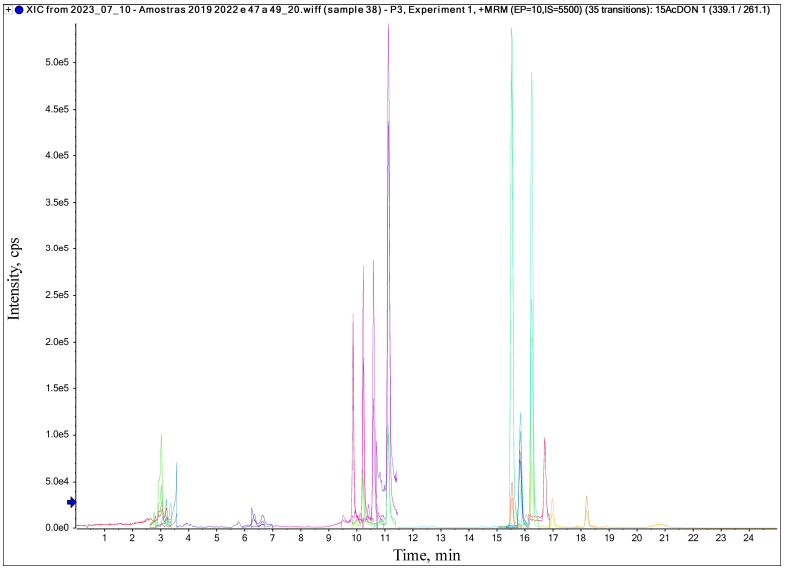


**Figure S3** - QTRAP 6500+ UHPLC/MS/MS ion chromatogram obtained from the selected reaction monitoring mode of a matrix-matched calibration sample fortified at an intermediate level (12 to 537 µg/kg).

**
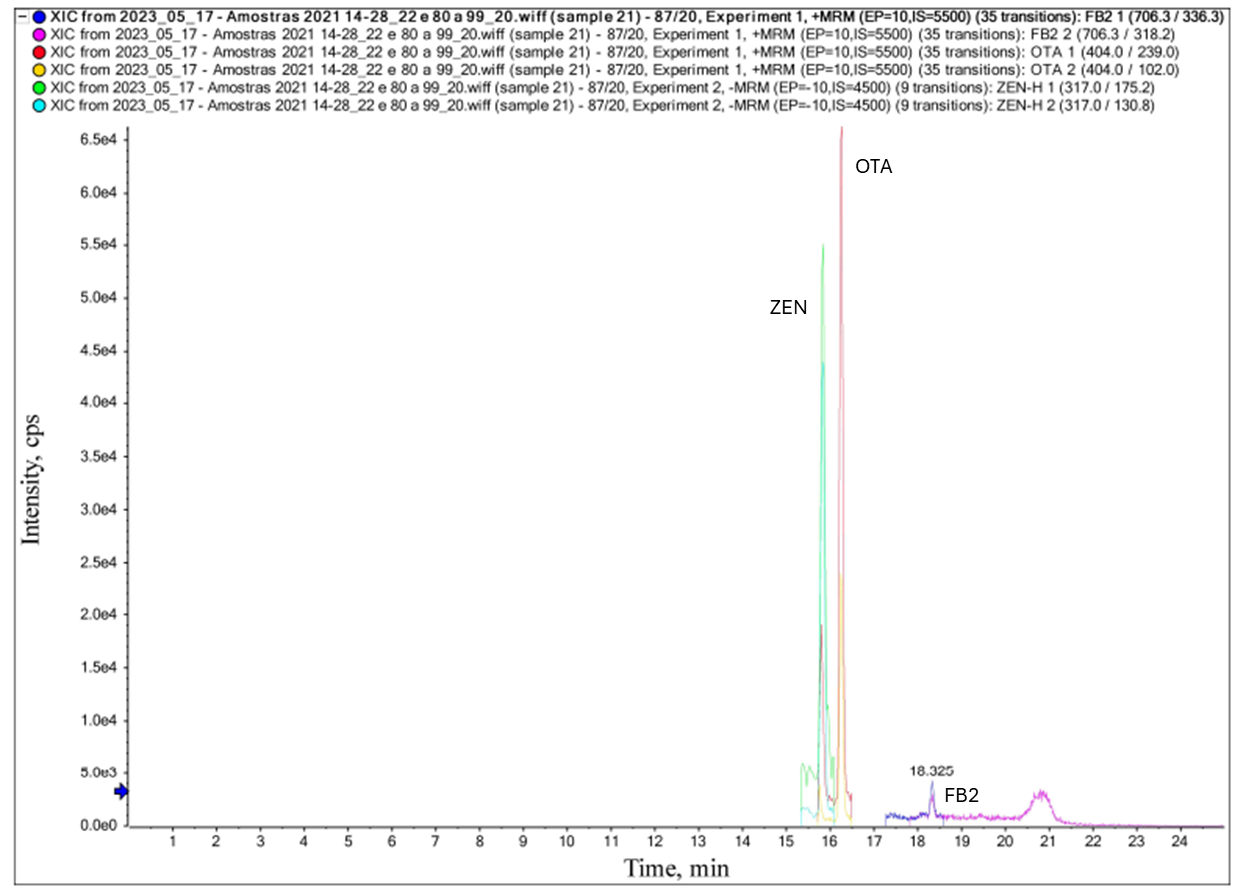
**

**Figure S4.** Chromatogram of a positive sample of Gotu kola (Hydrocotyle asiatica), showing the co-occurrence of zearalenone (9.2 µg/kg), ochratoxin A (11.6 µg/kg) and fumonisin B_2_ (traces).
